# Editorial: *in vitro* and *in vivo* non-clinical models of kidney cancers

**DOI:** 10.3389/fonc.2022.987682

**Published:** 2022-07-26

**Authors:** Valérian Dormoy, Odile Filhol, Carole Sourbier, Thierry Massfelder

**Affiliations:** ^1^ INSERM (French National Institute of Health and Medical Research) UMR-S1250, P3Cell, Université de Reims Champagne Ardenne, SFR CAP-SANTE, Reims, France; ^2^ INSERM (French National Institute of Health and Medical Research) UMR 1292-Interdisciplinary Research Institute of Grenoble (IRIG)-Biosanté, University Grenoble Alpes, CEA, Grenoble, France; ^3^ Division of Biotechnology Review and Research 1, Office of Biotechnology Products, Office of Pharmaceutical Quality, Center for Drug Evaluation and Research, United States Food and Drug Administration, Silver Spring, MD, United States; ^4^ INSERM (French National Institute of Health and Medical Research) UMR_S1260, Université de Strasbourg, Regenerative Nanomedicine, Centre de Recherche en Biomédecine de Strasbourg, Strasbourg, France

**Keywords:** kidney cancer, renal cell carcinoma (RCC), patient-derived xenograft (PDX), tumoroids, tissue slice culture, pre-clinical oncology, targeted therapy, immuno-oncology

Worldwide, kidney cancer accounts for 403 000 new cases and 175 000 deaths per year. Kidney tumors are highly heterogeneous at the molecular and genetic levels both intra- and inter-patients (1). Intrinsic and therapy-induced heterogeneity, and changes in the tumor microenvironment, might play a role in the development of resistance to therapies. To be reliable, nonclinical studies may require multiple models capable of recapitulating diverse aspects of the heterogeneity of the tumor and its microenvironment at different sites (i.e., primary and metastatic sites). There are no mouse transgenic models for kidney cancer that recapitulate the disease stages, despite many attempts to develop them (2–5). Well-characterized and controlled cancer models derived from kidney cancer patients (such as patient-derived xenografts, PDX) could become essential tools to understand how tumors evolved under therapeutic pressure and to identify potential mechanisms of resistance. *In vitro* models such as tumor slices in culture and cancer tissue fragments cultured with an air-liquid interface could provide clues about how the tumor cells interact with their microenvironment and how therapies might affect the immune populations present in the microenvironment. Traditional *in vitro* and *in vivo* models such as kidney cancer cell lines, spontaneous or induced tumors as well as cell-derived xenografts or xenografts of tumoroids derived from patients in immunocompromised rodents may also play a role in the nonclinical setting by allowing for higher throughput and consistency.

Diverse preclinical models used for kidney cancer were reviewed by Shapiro et al.. They comprehensively described the history, strengths, and limitations of 3-dimensional (3D) tumoroids, transgenic mouse models, and microphysiological devices, in addition to more traditional 2-dimensional (2D) cell cultures and PDX models, and critically evaluated their performance and ability to recapitulate tumors’ characteristics. The challenges and opportunities of pre-clinical kidney cancer models were also discussed by Pohl et al. in the context of drug development. Modelling of the complex ecosystem of kidney tumors will undoubtedly require the use of a combination of models and methodologies including artificial intelligence. Furthermore, Xiang et al. provided an overview of RCC drug resistance models that were developed over the last ten years. Numerous models have been used to study RCC drug resistance using both cell lines–derived and patient-derived *in vitro* and *in vivo* models, as well as 3D culture models investigating the relationship between hypoxia and TKI resistance in RCC. The authors also described transgenic mouse models generated using the CRISPR Cas-9 editing approach and their applications to explore the mechanisms of drug resistance in RCC. They concluded that all the models have advantages and drawbacks but are useful to better understand RCC drug resistance.

Recapitulation of kidney tumors’ characteristics is a key component for the establishment of relevant nonclinical models. Simon et al. tackled the transposability of *in vitro* results considering that kidney tumor cells in culture are known to generally behave differently than what is observed in situ, especially in terms of response to therapeutic compounds. They used a bulk (3’m)RNA sequencing approach to compare the transcriptomes of cells within tissues and cells in primary cultures. Changes in gene expression were observed for genes involved in DNA repair, cell cycle, hypoxia, metabolism, immune cell differentiation and cell adhesion. The study thus further reflected the adaptive changes of cells to the microenvironment, and clearly argued for a more reasonable selection of preclinical models. PDX models represent an invaluable tool for therapeutic evaluation, the study of drug resistance, and the identification of biomarkers. Their use for personalized medicine is however limited when considering the limited take rate of tumor growth at primo-implantation and the time of development (6). In their study, Gürgen et al. established and characterized a comprehensive panel of PDX models for renal cell carcinoma (RCC). The original feature of their cohort was the inclusion of metastatic sites as well as the development of PDX models from multiple regions for the same patients, representing, at least in part, the heterogeneity of RCC tumors. They performed an extensive molecular and drug response characterization. Such panel may help developing humanized PDX mouse models for preclinical testing of immune checkpoint inhibitors. Tumor-derived spheroids, known as tumoroids, play an increasingly important role in cancer research, but their use in the field of kidney cancer remains limited (7). In their report, Lugand et al. present new methods to develop this model from tumor tissue dissociation to tumoroid infiltration by immune cells. They also implemented an immune spheroid killing assay to analyse the effects of immune checkpoint inhibitors on tumoroids viability. Such model could potentially be standardized and validated to study and compare different immunotherapies. Tumor slices from freshly resected clear cell RCC were established as an *ex vivo* culture model to test antibodies against VISTA (also known as B7-H5, GI24, Dies1 and PD-1 homolog), as presented by Hong et al.. VISTA is a checkpoint molecule (8, 9) with an extracellular domain homologous to PD-L1 (10). The readout of this assay included the quantitative evaluation of cytokine production, proliferation, and cell death. Although the slices showed an increase in cell death overtime in culture conditions, the comparative analysis may indicate the antitumor efficacy of novel candidate molecular targets in individual patient-derived tumor tissues.

In conclusion, the domain of preclinical research is vast, complex, and should take into account all the tumors feature, including the tumors’ microenvironment and circulating tumor cells. It is also likely that lessons learned during the development and characterization of preclinical models for kidney tumors may translate to other cancer types. It would be beneficial for the scientific community to assemble in a publicly accessible repository of all the existing preclinical models to support the establishment of robust preclinical data, ultimately lowering the attrition rate (> 85%) of new potential therapeutic molecules in oncology.

## Author contribution

All authors have participated to the redaction of the manuscript and approved the final version of the manuscript.

## Conflict of interest

The authors declare that the research was conducted in the absence of any commercial or financial relationships that could be construed as a potential conflict of interest.

## Publisher’s note

All claims expressed in this article are solely those of the authors and do not necessarily represent those of their affiliated organizations, or those of the publisher, the editors and the reviewers. Any product that may be evaluated in this article, or claim that may be made by its manufacturer, is not guaranteed or endorsed by the publisher.

## Author disclaimer

This editorial reflects the views of the authors and should not be construed to represent US FDA’s views or policies.
